# Predictors of nonfunctional arteriovenous access at hemodialysis initiation and timing of access creation: A registry-based study

**DOI:** 10.1371/journal.pone.0181254

**Published:** 2017-07-27

**Authors:** Natalia Alencar de Pinho, Raphael Coscas, Marie Metzger, Michel Labeeuw, Carole Ayav, Christian Jacquelinet, Ziad A. Massy, Bénédicte Stengel

**Affiliations:** 1 Renal and Cardiovascular Epidemiology Team, CESP, INSERM U1018, Paris-Sud Univ, UVSQ, Paris Saclay University,Villejuif, France; 2 Division of Vascular Surgery, Ambroise Paré University Hospital, AP-HP, Boulogne-Billancourt, France; 3 Lyon-Sud University Hospital, Pierre-Bénite, France; 4 Epidémiologie et Evaluations Cliniques, Pôle S2R, CHRU Nancy, Nancy, France; 5 CIC-1433 Epidémiologie Clinique, Inserm, Nancy, France; 6 REIN Registry, Biomedicine Agency, Saint Denis, France; 7 Division of Nephrology, Ambroise Paré University Hospital, AP-HP, Boulogne-Billancourt, France; University of Washington, UNITED STATES

## Abstract

Determinants of nonfunctional arteriovenous (AV) access, including timing of AV access creation, have not been sufficiently described. We studied 29 945 patients who had predialysis AV access placement and were included in the French REIN registry from 2005 through 2013. AV access was considered nonfunctional when dialysis began with a catheter. We estimated crude and adjusted odds ratio (OR) with 95% confidence intervals (CI) of nonfunctional versus functional AV access associated with case-mix, facility characteristics, and timing of AV access creation. Analyses were stratified by dialysis start condition (planned or as an emergency) and comorbidity profile. Overall, 18% patients had nonfunctional AV access at hemodialysis initiation. In the group with planned dialysis start, female gender (OR 1.43, 95% CI 1.32–1.56), diabetes (OR 1.28, 95% CI 1.15–1.44), and a higher number of cardiovascular comorbidities (OR 1.27, 95% CI 1.09–1.49, and 1.31, 1.05–1.64, for 3 and >3 cardiovascular comorbidities versus none, respectively) were independent predictors of nonfunctional AV access. A higher percentage of AV access creation at the region level was associated with a lower rate of nonfunctional AV access (OR 0.98, 95% CI 0.98–0.99 per 1% increase). The odds of nonfunctional AV access decreased as time from creation to hemodialysis initiation increased up to 3 months in nondiabetic patients with fewer than 2 cardiovascular comorbidities and 6 months in patients with diabetes or 2 or more such comorbidities. In conclusion, both patient characteristics and clinical practices may play a role in successful AV access use at hemodialysis initiation. Adjusting the timing of AV access creation to patients’ comorbidity profiles may improve functional AV access rates.

## Introduction

Vascular access is a key factor in the prediction of outcomes of hemodialysis patients. Central venous catheter use is an independent predictor of infection, thrombosis, and hospitalization in this population [1,2]. It is also associated with unplanned dialysis start [3] and higher cost [4] compared with AV access (either fistulae or grafts). Importantly, patients starting hemodialysis through a catheter have higher mortality risk than patients who start with functional AV access [1,5–8]. Recommendations about vascular access thus agree that AV access is the best option for hemodialysis patients [9–11], and the Fistula First Catheter Last Initiative is a prominent example of efforts to improve vascular access outcomes [12].

Timely AV access referral is one of the most effective measures for preventing catheter use at hemodialysis initiation, but defining the ideal timing of AV access placement, notably of AV fistulae, remains challenging. Discrepancies in guidelines for vascular access reflect this problem. The European Best Practice Guidelines [9] recommend AV fistula creation at least 2 to 3 months before hemodialysis start, whereas the Kidney Disease Outcomes Quality Initiative [10] suggests at least 6 months. The Society for Vascular Surgery [11] defines a glomerular filtration rate of 20 to 25 ml/min/1.73m^2^ as the criterion for fistula referral, based on what it nonetheless described as “very low-quality evidence.” A population-based study conducted in Ontario, Canada, showed that only 40% of patients with predialysis AV fistula creation started hemodialysis within a time consistent with guideline recommendations (3 to 12 months), while 30% needed hemodialysis less than 3 months after AV fistula creation [13]. This study did not address the impact of timing on AV access functionality. Among the elderly in the United States, late AV access creation (1–3 months before hemodialysis initiation) was strongly associated with AV fistula failure. Early creation increased the odds of initiating hemodialysis through an AV fistula up to a time lag of 6–9 months. Beyond this lag, no improvement in fistula use rates was verified and the number of complication-related procedures increased [14].

Descriptions of determinants of nonfunctional AV access besides timing of AV access creation remain insufficient. Most epidemiological studies have assessed factors associated with AV access use *versus* catheter without distinguishing between patients who had AV access created before dialysis and those who did not. Women, older patients, and those with cardiovascular diseases are more likely to start hemodialysis with a catheter [7,15,16]. Poor Fewer visits to a nephrologist and the lack of erythropoiesis-stimulating agent (ESA) in the predialysis period have also been associated with catheter use at hemodialysis initiation [17]. Studies specifically comparing nonfunctional with functional AV accesses have found similar associations [5,14]. They were, however, restricted to the United States. Because of differences in patient selection and practices for predialysis AV access placement [18–20], nonfunctionality rates and their determinants may differ between Europe and North America. For instance, only 18% of US patients start hemodialysis with an AV access while this rate reaches 30 to 45% in Europe [5,6,21,22]. We therefore used data from the French REIN Registry to study the timing of AV access placement and determinants of nonfunctional AV access at hemodialysis initiation in a context of relatively high AV access use.

## Materials and methods

### Population

The French REIN registry includes all patients on chronic renal replacement therapy (RRT) for end-stage renal disease (ESRD)–either dialysis or transplantation. It began in 2002 and progressively expanded to include the entire country in 2011. Details on methods and quality control of the REIN registry have been described elsewhere [23]. In this study, we considered adult patients (age ≥18 years) who started hemodialysis from 2005 through 2013 (N = 64 407).

The REIN registry and its utilization for research purposes have been approved by the relevant French ethics committees, namely, the *Comité consultatif sur le traitement de l'information en matière de recherche* (CCTIRS) and the *Commission nationale de l'informatique et des libertés* (CNIL N° 903188). For population-based registries requiring exhaustiveness, French regulations do not require participants’ written or verbal informed consent. Patients are informed about the registration of all individuals with treated end-stage renal disease in the REIN registry by the nephrology clinic as well as about their right to not participate (opt out).

### Vascular access data and selection of AV access groups

**Fig 1:**
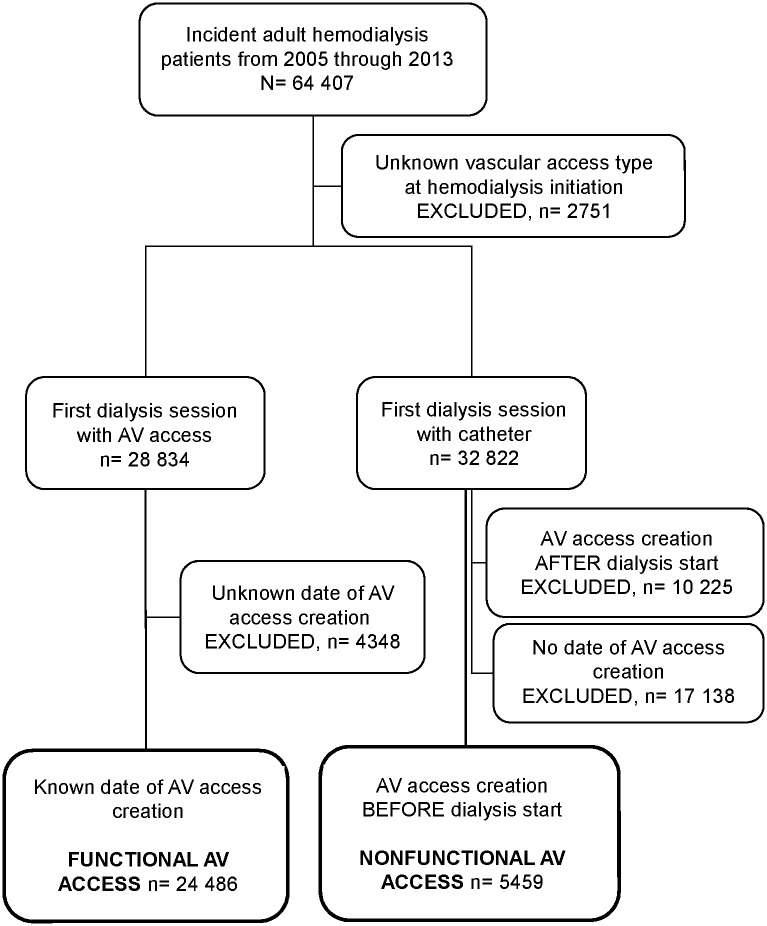
Cohort selection. Abbreviations: AV, arteriovenous.

After excluding patients with missing vascular access type at hemodialysis initiation (n = 2751), we classified the remaining patients according to their vascular access at hemodialysis initiation and by 2 data items (Fig 1): 1/ whether or not vascular access at the first dialysis session was a catheter; 2/ the date (day/month/year) of AV access placement, regardless of its functionality at dialysis initiation. There were two possible responses to the type of vascular access at the first hemodialysis session: catheter or other. When vascular access at the first hemodialysis session was not a catheter, patients were considered to have functional AV access, regardless of AV access date (n = 28 834, of whom 4348 were excluded because of missing dates). Of the 32 822 patients who started hemodialysis with a catheter, 10 225 had their first AV access creation after hemodialysis start and were not eligible for the study. Another 17 138 had no date of AV access placement and were also excluded. The remaining patients with AV access creation before and a catheter at hemodialysis onset were considered to have nonfunctional AV access (n = 5459).

Because the presence of AV access itself at hemodialysis start is not recorded, but only its date of creation, if any, we do not know how many patients among those with catheter access and no reported date did not have AV access and how many had a missing date for AV access creation. The percentage of missing dates is known, however, for patients who did start with functional AV access (15.1%). Patients with functional AV access not included in the analysis did not differ significantly from those included regarding gender, age, and emergency dialysis start. However, they stood out in particular for being less likely to have received predialysis erythropoiesis-stimulating agents (S1 Table). AV fistulae and AV grafts could not be distinguished at hemodialysis initiation, but in 2013 only 3% of prevalent patients in France had grafts [22].

### Other data

Patient-level data at baseline included date of RRT start, geographic region, age, gender, primary renal disease, body mass index (BMI), laboratory values over the 1-month period before RRT (serum albumin, hemoglobin level, and estimated glomerular filtration rate), comorbidities, predialysis use of ESA, and dialysis start condition (planned or emergency). Estimated glomerular filtration rate (eGFR) was calculated with the Modification of Diet in Renal Disease (MDRD) equation. Comorbidities included diabetes, number of cardiovascular diseases (among ischemic heart disease, stroke, peripheral vascular disease, heart failure, and dysrhythmia), lower limb amputation, malignancy, and mobility status as a proxy for frailty. Emergency dialysis start was defined in the registry as any first dialysis session initiated in life-threatening circumstances requiring dialysis within 24 h. Facility-level data included facility type (in-center dialysis unit, satellite unit, or self-dialysis) and facility ownership (public university, non-public university, private for-profit, or private not-for-profit). We calculated an annual percentage of patients with predialysis AV access by region and used it as a proxy for local availability and experience in vascular access surgery.

### Statistical analyses

Patient characteristics at baseline were described and compared between the 2 groups defined by AV access functionality at hemodialysis initiation, as described above. Categorical variables are presented as percentages and continuous variables as means ± standard deviations or medians (interquartile range), depending on the normality of their distributions. Chi-square, ANOVA, and Kruskal-Wallis tests were used as appropriate. Because of missing data for some variables (Table 1), we performed multiple imputation of 20 datasets with the fully conditional specification method [24,25]. The imputation model included all variables in Table 1, as well as geographic region, year of first ESRD treatment, and vital status at the end of the follow-up. Analyses through the 20 complete datasets were combined according to Rubin and Schencker’s rules [26]. We then estimated crude and adjusted odds ratios (OR) for nonfunctional *versus* functional AV access according to baseline characteristics. Because emergency dialysis start may modify the effect of covariates, we stratified these analyses by dialysis start condition, planned or as an emergency. To identify the optimal timing for AV access creation according to patient characteristics, we restricted the analysis to patients with a planned dialysis start. We assessed the percentage of nonfunctional AV access according to the timing of placement in 2 subgroups defined by patient comorbidity profile: no diabetes with fewer than 2 cardiovascular comorbidities *versus* diabetes or 2 or more cardiovascular disorders or both. Logistic regression was used to identify the shortest time between AV access placement and dialysis initiation associated with the lowest rate of nonfunctionality and to test interactions with patient characteristics.

**Table 1:** Cohort characteristics by arteriovenous access functionality status at hemodialysis initiation.

Characteristics	Functional AV access	Nonfunctional AV access	*P*-value	Imputed missing data
	n = 24 486 (%)	n = 5459 (%)		(%)
**Men**	65.0	58.4	<0.001	0
**Age** (years, median (IQR))	70.5 (59.6–78.9)	70.4 (58.4–78.6)	0.145	0
**Primary renal disease**			<0.001	0
Hypertensive/Vascular	27.6	26.2		
Diabetic nephropathy	23.3	30.9		
Glomerulonephritis	12.4	8.9		
Polycystic kidney disease	10.2	4.6		
Other	16.5	17.9		
Unknown	10.1	11.5		
**Diabetes**	39.2	49.5	<0.001	0.8
**Number of cardiovascular comorbidities**			<0.001	5.1
0	47.7	40.3		
1	25.8	26.1		
2	15.3	17.6		
3	7.9	10.9		
4 or 5	3.3	5.2		
**Lower limb amputation**	1.3	2.9	<0.001	5.1
**Malignancy**	8.2	9.8	<0.001	2.1
**Mobility status**			<0.001	11.1
Autonomous	87.9	79.5		
Needs assistance	9.4	15.0		
Totally dependent	2.7	5.5		
**Body mass index** (kg/m^2^)			<0.001	19.9
< 18.5	4.3	5.4		
[18.5–25.0]	40.0	37.8		
[25.0–30.0]	32.7	30.4		
≥30.0	23.0	26.4		
**Serum albumin** (g/l, mean ±SD)	35.1 (5.7)	32.5 (6.1)	<0.001	42.7
**Hemoglobin** (g/dl, mean ±SD)	10.6 (1.5)	10.1 (1.6)	<0.001	16.7
**Predialysis ESA treatment**	63.7	55.3	<0.001	9.6
**Estimated glomerular filtration rate** (MDRD ml/min/1.73m^2^)			<0.001	
eGFR≤5	7.8	12.8		
5<eGFR≤10	54.9	52.9		
10<eGFR≤15	29.1	25.6		
15<eGFR≤20	6.5	6.5		
eGFR>20	1.7	2.2		
**Emergency dialysis start**	9.6	37.2	<0.001	2.2
**Facility type**			<0.001	0
In center	92.2	97.0		
Satellite unit	4.2	1.5		
Self-dialysis	3.6	1.5		
**Facility ownership**			<0.001	0
Public university	18.2	27.3		
Public non-university	30.4	31.5		
Private for-profit	31.8	30.3		
Private not-for-profit	19.5	10.9		

Abbreviations: AV, arteriovenous; IQR, interquartile range; SD, standard deviation; ESA, erythropoiesis-stimulating agents; MDRD, Modification of Diet in Renal Disease.

Two-sided significance tests were used and *P*-values <0.05 were considered significant. Robust variance estimates for both logistic and Cox proportional hazard regressions were used to account for facility clustering effects. All statistical analyses were performed with SAS 9.4 (SAS Institute Inc, Cary, NC).

## Results and discussion

Among the 29 945 patients with available data who began hemodialysis from 2005 through 2013 after creation of a permanent vascular access, 18% had nonfunctional AV access at initiation. Table 1 summarizes patient characteristics according to AV access functionality status at the start of hemodialysis. Overall, median age was 70 (IQR, 58–79) years, 64% were men, 40% had diabetes and 27% at least 2 cardiovascular comorbidities; dialysis started as an emergency for 14%.

### Factors associated with nonfunctional AV access at hemodialysis initiation

Emergency first dialysis was strongly associated with nonfunctional *versus* functional AV access (crude OR 6.46, 95% CI 6.23–6.70). Because this dialysis start condition modified the effect estimates of other covariates, we stratified analyses by whether first dialysis was planned or an emergency. Among those with planned dialysis, nonfunctional AV access was significantly more frequent in women and in patients with diabetes, hypertensive or diabetic kidney disease, more cardiovascular comorbidities, lower limb amputation, malignancy, extreme eGFR or BMI values, lower serum albumin level, and anemia. In contrast, polycystic kidney disease and predialysis ESA treatment were associated with a lower likelihood of nonfunctional AV access. Patients with nonfunctional AV access started hemodialysis more often in in-center and public university facilities than their counterparts with functional AV access (Table 2). The higher a region’s annual percentage of patients with predialysis AV access, the lower its nonfunctional AV access rate at the start of dialysis. After multivariate adjustment, most associations remained similar, except that for age, which was reversed and became statistically significantly protective.

**Table 2:** Determinants of nonfunctional arteriovenous access in patients with a planned dialysis start (n^a^ = 25 570).

Variables	Crude OR (95% CI)	*P*-value	adjusted^b^ OR (95% CI)	*P*-value
**Women**	1.35 (1.25–1.45)	<0.001	1.43 (1.32–1.56)	<0.001
**Age** (10-year increase)	1.01 (1.01–1.01)	0.112	0.96 (0.96–0.97)	0.035
**Primary renal disease**		<0.001		<0.001
Hypertensive/ Vascular	1.37 (1.19–1.57)		1.25 (1.07–1.47)	
Diabetic nephropathy	1.90 (1.66–2.18)		1.33 (1.11–1.59)	
Glomerulonephritis	1		1	
Polycystic kidneys	0.71 (0.59–0.86)		1.01 (0.82–1.24)	
Other	1.52 (1.31–1.75)		1.43 (1.21–1.68)	
Unknown	1.58 (1.35–1.85)		1.35 (1.13–1.62)	
**Diabetes**	1.56 (1.45–1.67)	<0.001	1.28 (1.15–1.44)	<0.001
**Number of cardiovascular comorbidities**		<0.001		<0.001
0	1		1	
1	1.21 (1.1–1.32)		1.09 (0.98–1.21)	
2	1.27 (1.14–1.42)		1.10 (0.97–1.25)	
3	1.48 (1.29–1.70)		1.27 (1.09–1.49)	
4 or 5	1.67 (1.37–2.02)		1.31 (1.05–1.64)	
**Lower limb amputation**	2.21 (1.71–2.85)	<0.001	1.63 (1.21–2.20)	0.006
**Malignancy**	1.20 (1.06–1.36)	<0.001	1.13 (0.98–1.30)	0.099
**Mobility**		<0.001		<0.001
Autonomous	1		1	
Needs assistance	1.72 (1.53–1.93)		1.33 (1.16–1.52)	
Totally dependent	2.08 (1.71–2.53)		1.48 (1.19–1.85)	
**Body mass index** (kg/m^2^)		<0.001		0.034
< 18.5	1.25 (1.02–1.53)		1.09 (0.87–1.38)	
[18.5–25.0]	1			
[25.0–30.0]	1.02 (0.93–1.13)		1.10 (0.99–1.22)	
> = 30.0	1.26 (1.13–1.4)		1.22 (1.08–1.37)	
**Serum albumin** (1-g/l increase)	1.00 (1.15–0.87)	<0.001	0.96 (0.95–0.97)	<0.001
**Anemia** (hemoglobin < 10g/dl)	1.73 (1.59–1.87)	<0.001	1.23 (1.12–1.35)	<0.001
**Predialysis ESA treatment**	0.75 (0.69–0.81)	<0.001	0.80 (0.73–0.87)	<0.001
**Estimated glomerular filtration rate** (MDRD ml/min/1.73m^2^)		<0.001		<0.001
eGFR≤5	1.66 (1.43–1.92)		1.50 (1.26–1.78)	
5<eGFR≤10	1.06 (0.97–1.17)		1.11 (1.00–1.23)	
10<eGFR≤15	1		1	
15<eGFR≤20	1.06 (0.88–1.28)		1.01 (0.83–1.24)	
eGFR>20	1.41 (1.06–1.86)		1.36 (1.00–1.84)	
**Facility type**		<0.001		0.102
In center	1		1	
Satellite unit	0.41 (0.32–0.53)		0.72 (0.55–0.95)	
Self-dialysis	0.43 (0.33–0.57)		0.81 (0.61–1.09)	
**Facility ownership**		<0.001		0.077
Public university	1		1	
Public non-university	0.74 (0.67–0.82)		0.91 (0.82–1.02)	
Private for-profit	0.67 (0.61–0.74)		0.78 (0.70–0.88)	
Private not-for-profit	0.41 (0.36–0.46)		0.78 (0.67–0.91)	
**% Patients with AV access created, by region and by year** (1-% increase)	0.98 (0.98–0.98)	<0.001	0.98 (0.98–0.99)	<0.001
**Time from AV access creation to hemodialysis initiation** (months)		<0.001		<0.001
[0,1]	9.25 (8.17–10.47)		8.75 (7.68–9.96)	
[1,3]	1.66 (1.46–1.89)		1.53 (1.34–1.75)	
[3,6]	1.14 (0.98–1.31)		1.11 (0.96–1.29)	
[6,9]	1.05 (0.88–1.25)		1.03 (0.86–1.23)	
[9,12]	1.06 (0.87–1.29)		1.03 (0.84–1.27)	
≥12	1		1	

^a^ Mean number of patients with planned dialysis start through the 20 imputed data sets.

^b^ORs adjusted for year of hemodialysis initiation and region’s annual percentage of patients with predialysis arteriovenous access creation in addition to all variables in Table 1. Abbreviations: OR: odds ratio; CI: confidence interval; ESA, erythropoiesis-stimulating agents; MDRD, Modification of Diet in Renal Disease; AV, arteriovenous.

In patients with an emergency first dialysis, age, primary renal disease, diabetes, cardiovascular comorbidities, and the region’s annual percentage of patients with predialysis AV access were not significantly associated with nonfunctional AV access (S2 Table).

### Timing of AV access creation

**Fig 2:**
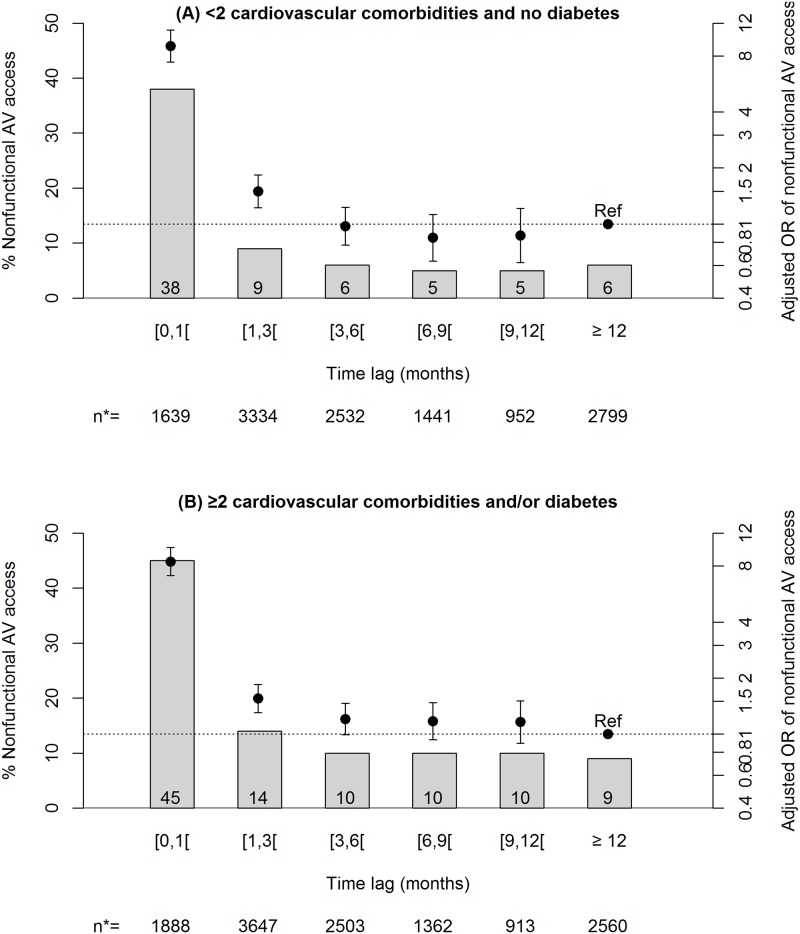
Prevalence and adjusted odds ratios of nonfunctional AV access at hemodialysis initiation according to timing of AV access creation and comorbidities in patients with a planned dialysis start. N* = number of patients within time lags through the 20 imputed data sets. Analyses were adjusted for all variables in Table 1 plus year of hemodialysis initiation and the region’s annual percentage of patients with predialysis AV access. *P*-value of interaction between comorbidity profile and timing of AV access creation: 0.059. *P*-value of timing of AV access creation in both stratified models: <0.001. Abbreviations: AV, arteriovenous; ref, reference.

In patients with planned dialysis start, the interval between AV access creation and hemodialysis initiation was <1 month for 14% of patients, 1–3 months for 27%, 3–12 months for 38%, and >12 months for 21%. The median interval (IQR) was 4.6 (2.0–10.9) months for the group with functional AV access and 1.3 (0.3–4.8) months for the group with nonfunctional AV access. When the time lag between AV access creation and hemodialysis initiation was <30 days, the prevalence of nonfunctional AV access was 38% in the relatively healthy subset of patients, i.e., those without diabetes and 1 or no cardiovascular comorbidities, while it was 45% in the group of patients with diabetes, or ≥2 cardiovascular conditions, or both (Fig 2). The lowest prevalence observed was 5% for the first subgroup, when AV access was placed ≥6 months predialysis. The lowest rate in the second subgroup was 9%, when AV access was created up to 12 months predialysis. The adjusted odds ratios for nonfunctional AV access decreased as time from creation to hemodialysis initiation increased up to 3 months in the healthier subgroup (*P* <0.001) and 6 months in the less healthy (*P* 0.058); they stabilized thereafter (*P*-value for interaction between comorbidity profile and timing of AV access creation 0.059). Sex and age did not interact significantly with the timing of AV access creation (*P*-values of 0.147 and 0.366, respectively).

This study shows that during the study period, a significant portion of patients in France started hemodialysis through a central venous catheter despite previous AV access placement. While several patient characteristics, such as gender and comorbidities, were independent predictors of successful AV access use at hemodialysis initiation, the factors most strongly associated with nonfunctional AV access were health care-related: late AV access creation and emergency first dialysis. Interestingly, a higher rate of AV access creation at the region level was associated with a lower likelihood of nonfunctional AV access. Our results also highlight the importance that the patient’s comorbidity profile may have in determining the optimal timing for predialysis AV access creation.

We identified a number of factors associated with nonfunctional AV access among incident hemodialysis patients. Nonmodifiable factors, such as gender and chronic diseases, are well-established determinants of AV access use and patency. For instance, previous studies have showed that women are less likely to have an AV access created before dialysis [7,27], to start hemodialysis with an AV fistula [16], to have an AV fistula rather than an AV graft as vascular access [17], and to have an adequate AV fistula blood flow for chronic dialysis [28]. In our study, women had a significantly and independently higher odds of nonfunctional AV access. This relation has also been described in the elderly hemodialysis population in the US [14]. The reasons for these associations remain unknown. Studies assessing the role of vein diameter in the relation between gender and AV fistula maturation are not conclusive [28,29]. Surprisingly, we found that age was inversely associated with nonfunctional AV access after adjustment for the other covariates. Previous reports have showed less frequent AV fistula creation [7], poorer AV fistula maturation [30], and more long-term complications of vascular access [15] in the elderly. We believe that the relation seen between nonfunctional AV access and age in our study may partly reflect selection for AV access creation in elderly patients. Older patients may also be more likely to receive AV grafts and AV fistulae at the elbow, which has been associated with higher rates of AV access use at hemodialysis initiation and patency [31–34].

The higher odds of nonfunctional AV access in public university hospitals compared with non-university and private hospitals (both for- and not-for-profit) were only partly explained by case-mix, but we cannot rule out the influence of unmeasured confounders. The percentage of patients with an AV access created before dialysis at the regional level was also associated with nonfunctional AV access. The more patients in a region who had an AV access created predialysis, the fewer patients who started hemodialysis with nonfunctional AV access. This finding was stable for changes in both the variable type (continuous or tertiles) and the geographic unit used in variable construction (French regions or districts). It suggests that structural aspects of health care may play a role in AV access functionality. Further investigation is necessary to ascertain the particular aspect of health care organization (referral for AV access creation, or supply of vascular surgery) that is failing to achieve AV access goals in some contexts. A survey of vascular access experts from 37 European countries reported some possible structural barriers to adoption of fistula-first policy: limited access to surgical resources, lack of training, and low use of preoperative diagnostic imaging of vessels [35]. Emergency dialysis start, one of the strongest predictors of nonfunctional AV access in our study, is another factor that is potentially modifiable to improve permanent vascular access use at hemodialysis initiation. Previous studies have shown that dialysis start condition is associated with late referral [36,37], quality of chronic kidney disease care [38], and patient reluctance in preparing for RRT [39]. Because of interactions with “emergency HD start” in the relations between patient characteristics and the functional status of vascular access, we conducted separate analyses for patients with and without emergency hemodialysis start. Comparison of the findings between Table 2 and S2 Table shows clearly that associations with gender and several comorbidities differ between the two groups.

Because the timing of permanent vascular access creation is an important and potentially modifiable risk factor for nonfunctional AV access at hemodialysis initiation, we sought to identify an optimal time lag between creation and dialysis start. We found that creation 3 months before dialysis seemed sufficient to produce the lowest rate of nonfunctional AV access in patients without diabetes and with 1 or no cardiovascular comorbidity. In contrast, for patients with either diabetes or at least 2 cardiovascular conditions or both, the lowest rate required AV access placement at least 6 months predialysis. These findings are consistent with those described for elderly patients in an analysis from the United States Renal Data System (URSDS) [14], although that study did not report testing interactions between subgroups. They suggest that time to successful AV access use, taking into account the intercurrent events that may precipitate dialysis start or delay AV access maturation, may depend on the patient’s comorbidity profile. This point is particularly relevant as current guidelines for AV access creation do not provide customized recommendations according to patient characteristics [9–11].

Nevertheless, anticipating hemodialysis onset remains challenging because of the nonlinearity of CKD progression. A strategy of closer follow-up of patients eligible for an AV fistula and the choice of new-generation grafts allowing early cannulation may facilitate determination of the ideal timing for AV access creation [40]. Optimal timing should also involve the avoidance of AV access creation in patients who will not undergo dialysis. More investigation is thus needed to identify timely creation, especially in patients with numerous comorbidities. A decision analysis assessing the ideal timing for AV fistula referral through Monte Carlo simulation found that earlier referral for older patients had virtually no impact on rates of successful AV access use at hemodialysis initiation, but increased the frequency of unnecessary AV access creation [41]. This finding was due mainly to mortality before hemodialysis initiation, which suggests that early AV access creation in patients with a high comorbidity burden may often be futile. However, a population-based study conducted in Canada [13] showed that patients with a higher Deyo-Charlson Comorbidity Index were more likely to start hemodialysis after AV fistula creation, when age, sex and the competing risk of death are taken into account.

Almost one patient in five had a nonfunctional AV access at hemodialysis initiation in our study. Although this rate is substantial, it is appreciably lower than those reported in Canada (33%) [7] or the United States (50%) [5,6]. Underestimation of nonfunctional AV access prevalence in our study due to missing data may contribute in part to this difference. However, international comparisons by the Dialysis Outcomes and Practice Patterns Study (DOPPS) have shown differences in patient characteristics and in clinical practices between Europe and North America that may also explain this discrepancy. Hemodialysis patients in North America have higher prevalence rates of obesity, diabetes, and cardiovascular comorbidities than those in Europe [42]. These comorbidities were independent predictors of nonfunctional AV access in our analysis. Not only are rates of AV access at hemodialysis initiation in Europe substantially higher than in North America [18,43], but surgical training has been shown to be more intensive in Europe [44]. Earlier AV fistula cannulation in European countries [18] may also partly explain the lower prevalence of nonfunctional AV access in our study, as evidenced by the high percentage of successful AV access use at initiation among patients whose AV access was created less than 1 month predialysis (62 to 55%, according to patient profile, Fig 2).

Major strengths of this study include the size and unselected nature of our registry-based population, which enable generalization of our results to all French patients on hemodialysis. In addition, we were able to take major potential confounders into account in the analyses, including whether dialysis started on a planned or emergency basis. We showed that initiation was unplanned for more than one third of the patients with nonfunctional AV access and that emergency start modified OR estimates for comorbidities associated with nonfunctional AV access.

Our study also has limitations. First, because of their observational nature, results indicate associations rather than causes and should be interpreted with caution. Second, we excluded patients with missing dates for AV access creation from the analyses. In view of the characteristics of patients with functional AV access not included in the analyses, including but not limited to extreme eGFR levels and lack of predialysis ESA treatment, the prevalence of nonfunctional AV access in our study may be underestimated. Nevertheless, the simulation of missing dates on the prevalence estimate showed that their potential impact would be low; for example, if the missing date rate in this group was twice (30%) that in the group with function AV access, the prevalence of nonfunctional AV access would rise from 18% only to 21%. A recent study from the French national health insurance database (covering 77% of the French population) showed that 30.3% of the patients starting hemodialysis had an arteriovenous fistula created within 1 to 12 months before initiation in 2013 [45]. In our study, for the same period, 29.4% of patients starting dialysis had undergone predialysis AV access creation. Most missing dates of AV access creation at baseline may thus correspond to a lack of AV access placement before hemodialysis initiation. Finally, the registry does not record the number of predialysis AV access placements, type (fistulae or grafts), their precise location, and other potential determinants of AV access functionality. Smaller studies have suggested that vein diameter [46], surgical experience [44], and patient behavior [47] may influence vascular access use and patency. In particular, the type of AV access may have a major impact on its functionality at hemodialysis initiation, with fistulae requiring a longer maturation period after creation. Detailed information about AV access creation is currently being collected in the Chronic Kidney Disease–Renal Epidemiology Information Network Study (CKD-REIN), a large cohort of patients with moderate or advanced chronic kidney disease and should make it possible to answer these questions [48].

## Conclusion

A substantial percentage of patients, particularly women and those with comorbidities, have nonfunctional AV access at hemodialysis initiation in France. Late or irregular nephrological care may, however, be the most important determinant of nonfunctional AV access use at hemodialysis initiation, through its association with delayed AV access creation and emergency hemodialysis start. Conversely, a higher rate of AV access creation at the regional level was an independent predictor of successful AV access use. Moreover, adjusting the timing of AV access creation to each patient’s comorbidity profile may improve functional AV access rates at initiation.

## Supporting information

S1 Table Comparison between patients with functional arteriovenous access at hemodialysis initiation included and not included in the study. (DOCX)

S2 Table Predictors of nonfunctional arteriovenous access in patients with emergency dialysis start. (DOCX)
