# Supplementary material for: Predictors of nonfunctional arteriovenous access at hemodialysis initiation and timing of access creation: A registry-based study
Source: PLoS One. 2017 Jul 27;12(7):e0181254. doi: 10.1371/journal.pone.0181254 (PMC5531527; doi:10.1371/journal.pone.0181254)
Supplement: S1 Table — (DOCX) [file pone.0181254.s001.docx]

S1 Table. Comparison between patients with functional arteriovenous access at hemodialysis initiation included and not included in the study.

| **Characteristics** | **Included** | **Not included** | ***P*-value** |
| --- | --- | --- | --- |
|  | n=24 486 (%) | n=4348 (%) |  |
| **Men** | 65.0 | 64.7 | 0.758 |
| **Age** (years, median (IQR)) | 70.4 (58.4-78.6) | 70.1 (57.9-79) | 0.094 |
| **Primary renal disease** | |  | <0.001 |
| Hypertensive/Vascular | 27.6 | 26.3 |  |
| Diabetic nephropathy | 23.5 | 23.3 |  |
| Glomerulonephritis | 12.4 | 9.2 |  |
| Polycystic kidney disease | 10.2 | 7.1 |  |
| Other | 16.5 | 15.5 |  |
| Unknown | 10.1 | 18.4 |  |
| **Diabetes** | 39.2 | 37.7 | 0.070 |
| **Number of cardiovascular comorbidities** | | | <0.001 |
| 0 | 47.7 | 55.6 |  |
| 1 | 25.8 | 22.8 |  |
| 2 | 15.3 | 13.2 |  |
| 3 | 7.9 | 6.0 |  |
| 4 or 5 | 3.3 | 2.3 |  |
| **Lower limb amputation** | 1.3 | 1.2 | 0.607 |
| **Malignancy** | 8.2 | 8.4 | 0.697 |
| **Mobility status** |  |  | <0.001 |
| Autonomous | 87.9 | 83.7 |  |
| Needs assistance | 9.4 | 11.9 |  |
| Totally dependent | 2.7 | 4.5 |  |
| **Body mass index** (kg/m²) | |  | <0.001 |
| < 18.5 | 4.3 | 6.2 |  |
| [18.5-25.0[ | 40.0 | 41.9 |  |
| [25.0-30.0[ | 32.7 | 31.4 |  |
| ≥30.0 | 23.0 | 20.5 |  |
| **Serum albumin** (g/l, mean ±SD) | 35.1 (5.7) | 34.4 (6) | <0.001 |
| **Hemoglobin** (g/dl, mean ±SD) | 10.6 (1.5) | 10.4 (1.7) | <0.001 |
| **Predialysis ESA treatment** | 63.7 | 32.2 | <0.001 |
| **Estimated glomerular filtration rate** (MDRD ml/min/1.73m²) | | | <0.001 |
| eGFR≤5 | 7.8 | 12.3 |  |
| 5<eGFR≤10 | 54.9 | 46.5 |  |
| 10<eGFR≤15 | 29.1 | 30.2 |  |
| 15<eGFR≤20 | 6.5 | 8.6 |  |
| eGFR>20 | 1.7 | 2.4 |  |
| **Emergency dialysis start** | 9.6 | 9.9 | 0.504 |
| **Facility type** |  |  | <0.001 |
| In center | 92.2 | 91.9 |  |
| Satellite unit | 4.2 | 3.5 |  |
| Self-dialysis | 3.6 | 4.6 |  |
| **Facility ownership** | |  | <0.001 |
| Public university | 18.2 | 17.4 |  |
| Public non-university | 30.4 | 23.6 |  |
| Private for-profit | 31.8 | 47.1 |  |
| Private not-for-profit | 19.5 | 11.8 |  |

Abbreviations: AV, arteriovenous;  IQR, interquartile range; SD, standard deviation; ESA, erythropoiesis-stimulating agents; MDRD, Modification of Diet in Renal Disease.
