# Supplementary material for: Predictors of nonfunctional arteriovenous access at hemodialysis initiation and timing of access creation: A registry-based study
Source: PLoS One. 2017 Jul 27;12(7):e0181254. doi: 10.1371/journal.pone.0181254 (PMC5531527; doi:10.1371/journal.pone.0181254)
Supplement: S2 Table — (DOCX) [file pone.0181254.s002.docx]

S2 Table. Predictors of nonfunctional arteriovenous access in patients with emergency dialysis start (n^a^= 4375).

| **Variables** | **Adjusted^b^ OR (95% CI)** | ***P*-value** | ***P*-value for interaction with dialysis start condition** |
| --- | --- | --- | --- |
| **Women** | 1.30 (1.13-1.51) | 0.001 | 0.444 |
| **Age** (10-year increase) | 0.94 (0.93-0.94) | 0.057 | 0.029 |
| **Primary renal disease** | | 0.065 | 0.021 |
| Hypertensive/ Vascular | 1.12 (0.86-1.47) |  |  |
| Diabetic nephropathy | 1.22 (0.91-1.63) |  |  |
| Glomerulonephritis | 1 |  |  |
| Polycystic kidneys | 0.85 (0.56-1.28) |  |  |
| Other | 1.35 (1.02-1.79) |  |  |
| Unknown | 1.41 (1.03-1.92) |  |  |
| **Diabetes** | 0.97 (0.79-1.17) | 0.734 | <0.001 |
| **Number of cardiovascular comorbidities** | | 0.683 | 0.003 |
| 0 | 1 |  |  |
| 1 | 0.89 (0.74-1.08) |  |  |
| 2 | 0.89 (0.73-1.09) |  |  |
| 3 | 0.92 (0.73-1.17) |  |  |
| 4 or 5 | 0.98 (0.72-1.33) |  |  |
| **Lower limb amputation** | 1.80 (1.16-2.79) | 0.007 | 0.537 |
| **Malignancy** | 1.14 (0.90-1.45) | 0.277 | 0.779 |
| **Mobility** |  | 0.244 | 0.076 |
| Autonomous | 1 |  |  |
| Needs assistance | 1.19 (0.97-1.46) |  |  |
| Totally dependent | 1.15 (0.86-1.53) |  |  |
| **Body mass index** (kg/m²) | | 0.051 | 0.566 |
| < 18.5 | 1.27 (0.89-1.82) |  |  |
| [18.5-25.0[ | 1 |  |  |
| [25.0-30.0[ | 1.11 (0.93-1.32) |  |  |
| >=30.0 | 1.30 (1.07-1.57) |  |  |
| **Serum albumin** (1-g/l increase) | 0.97 (0.95-0.98) | <0.001 | 0.164 |
| **Anemia** (hemoglobin < 10g/dl) | 1.13 (0.97-1.31) | 0.145 | 0.393 |
| **Predialysis ESA treatment** | 0.78 (0.68-0.90) | 0.001 | 0.698 |
| **Estimated glomerular filtration rate** (MDRD ml/min/1.73m²) | | 0.143 | 0.690 |
| eGFR≤5 | 1.34 (1.04-1.72) |  |  |
| 5<eGFR≤10 | 1.20 (1.01-1.43) |  |  |
| 10<eGFR≤15 | 1 |  |  |
| 15<eGFR≤20 | 1.02 (0.75-1.38) |  |  |
| eGFR>20 | 1.25 (0.77-2.03) |  |  |
| **Facility type** |  | 0.266 | 0.647 |
| In center | 1 |  |  |
| Satellite unit | 0.58 (0.29-1.15) |  |  |
| Self-dialysis | 0.80 (0.44-1.45) |  |  |
| **Facility ownership** | | 0.354 | 0.946 |
| Public university | 1 |  |  |
| Public non- university | 0.73 (0.61-0.89) |  |  |
| Private for-profit | 0.63 (0.49-0.82) |  |  |
| Private not-for-profit | 0.85 (0.71-1.03) |  |  |
| **% Patients with an AV access created by region and by year** (1% increase) | 0.99 (0.99-1.00) | 0.008 | 0.003 |
| **Time from AV access creation to hemodialysis initiation** (months) | | <0.001 | 0.470 |
| [0,1[ | 8.07 (6.54-9.97) |  |  |
| [1,3[ | 1.78 (1.45-2.19) |  |  |
| [3,6[ | 1.03 (0.82-1.31) |  |  |
| [6,9[ | 1.32 (1.00-1.73) |  |  |
| [9,12[ | 1.12 (0.82-1.53) |  |  |
| ≥12 | 1 |  |  |

^a^ Mean number of patients with planned dialysis start through the 20 imputed data sets. ^b^ORs adjusted for year of hemodialysis initiation and region’s annual percentage of patients with predialysis arteriovenous access creation in addition to all variables in Table 1. Abbreviations: OR: odds ratio; CI: confidence interval; ESA, erythropoiesis-stimulating agents; MDRD, Modification of Diet in Renal Disease; AV, arteriovenous.
